# Use of COVID-19 Test Positivity Rate, Epidemiological, and Clinical Tools for Guiding Targeted Public Health Interventions

**DOI:** 10.3389/fpubh.2022.821611

**Published:** 2022-03-16

**Authors:** Nivedita Gupta, Salaj Rana, Samiran Panda, Balram Bhargava

**Affiliations:** Indian Council of Medical Research, New Delhi, India

**Keywords:** COVID-19, bed capacity, case to infection ratio, epidemiology, infection to hospitalization ratio, public health, test positivity rate

## Abstract

India experienced a second wave of COVID-19 infection with an unprecedented upsurge in the number of cases. We have analyzed the effect of different restrictive measures implemented in six Indian states. Further, based on available national and international data on disease transmission and clinical presentation, we have proposed a decision-making matrix for planning adequate resources to combat the future waves of COVID-19. We conclude that pragmatic and well calibrated localized restrictions, tailored as per specific needs may achieve a decline in disease transmission comparable to drastic steps like national lockdowns. Additionally, we have underscored the critical need for countries to generate local epidemiological, clinical and laboratory data alongwith community perception and uptake of various non-pharmaceutical interventions, for effective planning and policy making.

## Introduction

India experienced a second wave of COVID-19 infection which peaked in April-May 2021. Rise in cases across the country was associated with emergence and spread of the delta variant of SARS-CoV-2, which is known to be highly transmissible with possibly enhanced disease severity (1–3). Non-pharmaceutical interventions (NPIs) including masking, social distancing, large-scale lockdowns, resource allocation, risk communication and travel restriction have played an important role in reducing transmission of SARS-CoV-2 worldwide (4). Nations such as Europe, China, Japan and United States of America implemented different combinations of NPIs to prevent local epidemics and reduce the burden on healthcare systems (5–7). Based on global learnings and feasibility of implementing various NPIs, effective strategies for interrupting disease transmission during the second wave of COVID-19 infection were considered in India. Multiple public health interventions were deployed for containment, reducing disease severity, and strengthen healthcare infrastructure. Major stakeholders worked in tandem to expeditiously augment hospital bed capacity, ventilators, oxygen supplies, personal protective equipment and availability of essential drugs & diagnostics. Vaccination of target population was upscaled, despite challenges of vaccine hesitancy and high levels of disease transmission. Despite these efforts, the healthcare infrastructure was overwhelmed with an unprecedented public health crisis during the peak of second wave. In this paper, we have highlighted the wide variation of COVID-19 infection trends across different districts and states of India. This is possibly the first analysis of its kind where whole national testing database has been used to analyze the effect of decentralized restrictive measures implemented in different parts of India during the second wave of infections. In addition, we have also proposed a matrix for health resource allocation to better deal with localized outbreaks of SARS-CoV-2 in future.

## Considering an Effective NPI for Reducing Disease Transmission: Nationwide Lockdown Vis-à-vis Local Data-Based Interventions at District Level

Nation-wide lockdown was suggested as an immediate step to reduce disease transmission during peak of the second wave in India. However, the social and economic implications were reviewed vis-à-vis slowing down disease spread by this strategy. India had earlier imposed four National lockdowns, which lasted from March 24 to May 31, 2020, followed by gradual re-opening. This approach had reasonably flattened the curve of the first wave of COVID-19 with its peak in September 2020 (8). Though the mortality was reduced but at the expense of depletion of economy, industry and unemployment (9). An estimated number of 230 million individuals slipped below minimum wage poverty line (10). Micro, small and medium enterprises (MSMEs), which account for 30% of the gross domestic product (GDP) were shut down due to inadequate supply of raw materials, crisis of workers and funds. Migrant workers, constituting about 20% of the workforce lost employment and had to return to their native places (11). Adverse impact of a future national lockdown was speculated to outweigh its benefits and therefore the option was not prioritized (9–13). The central government gave a generic advice to states to use test positivity rate (TPR) alongwith locally available data related to disease transmission and hospitalization for imposing or easing restrictions. All states were also advised to enforce and continue other proven NPIs, for disease control. Due to federal structure of India and health being a state subject, adaptation of the central guidance has varied across states, which have adopted different extent of NPIs, varying from generalized to localized measures. In-depth deliberations were undertaken by the national and state level Task Forces on COVID-19 to carve a decision matrix for imposing localized restrictions with effective outcomes.

## Restrictive Measures Implemented in India During the Second Wave of COVID-19 Infection

**Figure 1:**
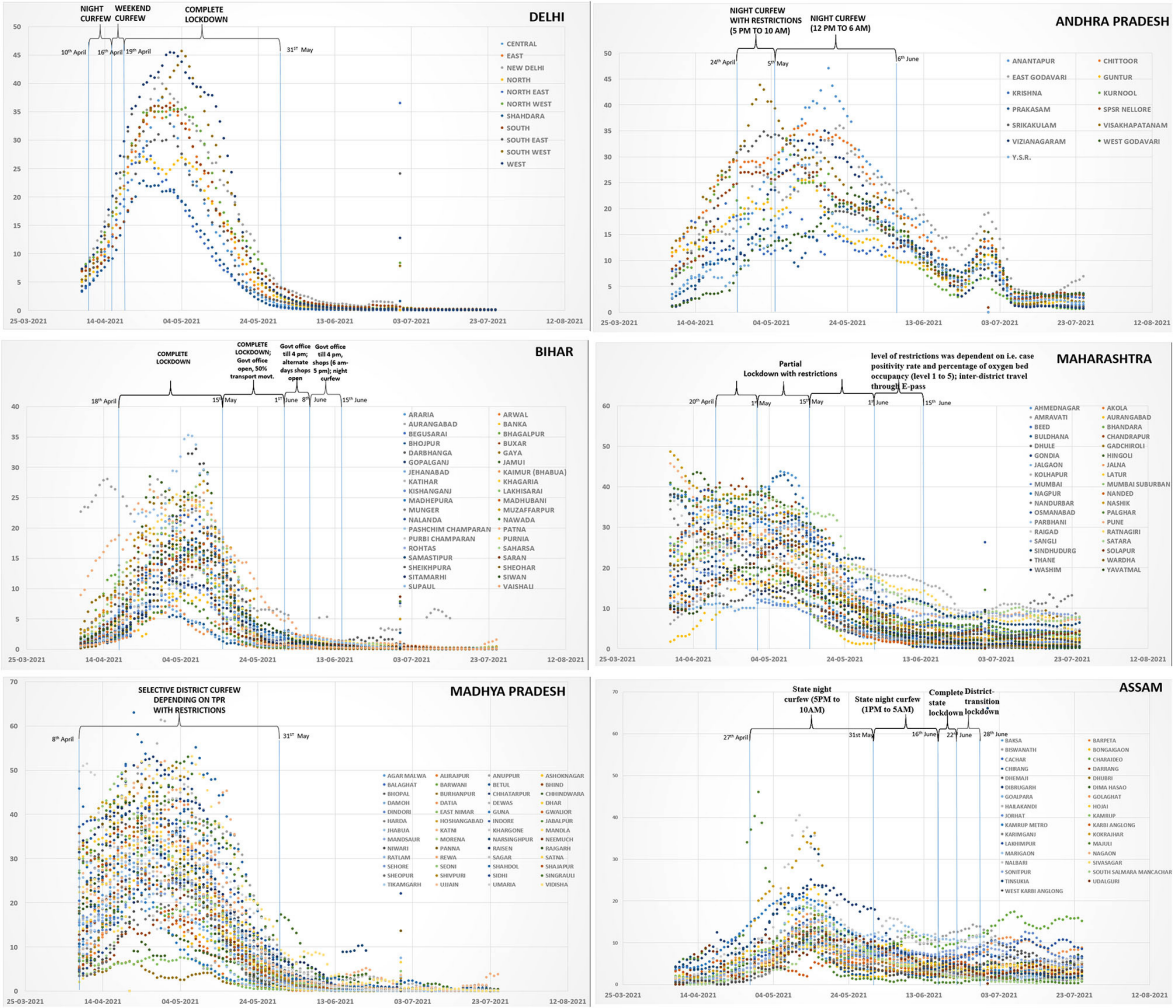
Types of Lockdown interventions and trends in six states of India (Delhi, Andhra Pradesh, Bihar, Maharashtra, Madhya Pradesh, and Assam). X-axis depicts the dates and y-axis shows the rolling average of test positivity rates.

The localized restrictive measures implemented in India during the second wave of COVID-19 infection were heterogeneous in nature and varied amongst states. Broadly three major types of restrictions were imposed by states: complete, partial and mixed. We have analyzed the decline in COVID-19 TPR between April 15th and July 31st, 2021 vis-à-vis the type of restrictive measures in six geographically representative states/union territories of India, to understand the effectiveness of different types of restrictions (14) (Figure 1). In North India, Delhi implemented night curfew for a week, starting from April 10th, 2021, followed by one weekend curfew and a subsequent complete lockdown from April 19th to May 31st, 2021. In Eastern India, the state of Bihar implemented a lockdown strategy which was very similar to Delhi and continued from April 18th to June 15th, 2021. In North-Eastern part, the state of Assam imposed a night curfew on April 27th, 2021 in the whole state, followed by graded restrictions and relaxations in districts based on their weekly TPR. This continued till end of June 2021. In Central India, Madhya Pradesh implemented a district-centric restrictive strategy from April 8th to June 30th, 2021 with strict monitoring of restrictions/relaxations based on weekly TPR. In West, Maharashtra imposed a partial lockdown starting from April 20th, 2021 till mid of June 2021, which was based on regional TPR and oxygen bed occupancy. In Southern India, the state of Andhra Pradesh implemented a partial lockdown between April 24th and June 10th, 2021 starting as a night curfew, extended to late morning and then afternoon hours. During our period of observation from April 15th to July 31st, 2021, the states of Andhra Pradesh, Maharashtra and Assam took a total of 81, 56, and 77 days respectively to attain an overall TPR of <5%. But even after this time, some of the districts in these states continuously reported a TPR of >5% and even >10% (Figure 1). Andhra Pradesh even witnessed another upsurge in infections within less than a month's time after the restrictions were relaxed. In Delhi, Bihar and Madhya Pradesh, the TPR declined to <5% in a duration of 40, 35, and 41 days respectively. All the three states continued to report low TPR in all districts, till the end of our observation period upto July 31st, 2021. Madhya Pradesh implemented district specific restrictive measures wherever a weekly TPR of >5% was reported. A complete lockdown was implemented in districts with >10% TPR and no inter-state or inter-district travel was permitted, other than for accessing essential services. Delhi and Bihar achieved a TPR of <5% only after complete lockdown. However, outcome of the strategy implemented in Madhya Pradesh in terms of reduction of TPR to <5%, appeared to be comparable with Bihar and Delhi with the added advantage of minimum disruption of services. From mid-April to May end, the overall 7-day average test positivity rate of Madhya Pradesh, a state that implemented only district centric restrictions, declined from 23 to 3% against the National average of 9.5% on May 31st, 2021. We could not analyze the effect of other NPIs that may have contributed to this observation. Despite varying dates of lockdown initiation and end in different districts, it was observed that there was a gradual overall decline in TPR toward the later phase of the lockdown.

## Testing Infrastructure in India

In India molecular and rapid antigen lateral flow assays are the mainstay of diagnosis of COVID-19 with a per test cost of $2 and 1, respectively. The country has a network of more than 3,000 molecular testing laboratories with 44 and 56% laboratories in the public and private sector respectively. Cost of testing in public sector is borne by the government whereas private sector charges from the clients. Approximately 75% of the molecular testing is undertaken at the public laboratories. The National COVID-19 testing database is centrally situated, managed by the Indian Council of Medical Research (ICMR) and captures country-wide testing results of all 734 districts in India. Till date, more than 700 million cumulative COVID-19 tests have been conducted with a National daily average of 1,000–1,400 tests per million, much more than the recommended WHO average of 140 tests per million population per day for a TPR of <5% (15). During the peak of second wave of infections, all 37 states/UTs in India had conducted much more than 140 COVID-19 tests per million per day (Supplementary Material). Delhi, Bihar, Assam, Madhya Pradesh, Maharashtra, and Andhra Pradesh reported an average TPM of 3,544, 955, 1,187, 665, 2,027, and 1,114 from April 15th to May 31st, 2021. The national daily average was 1997 during the same time (Supplementary Material).

## Using Test Positivity Rates to Impose/Ease Relaxations

Strength of the centralized testing database at ICMR was leveraged to impose or ease restrictions and whole national dataset was used for analysis. From mid-April 2021, an innovative strategy of rolling average of weekly district test positivity was adopted as a guiding tool to instate/ease restrictive measures and prioritize public health interventions in various districts. For districts reporting <5% TPR, restrictions were relaxed, sentinel testing was undertaken for suspect patients and close contacts, high risk individuals, outdoor and in-patients, cases of influenza like and severe acute respiratory illness, contact tracing, case-based management and health care infrastructure were maintained with varying implementation patterns. For districts reporting TPR between 5 and 10%, partial restrictions were imposed, and testing was upscaled. Contact tracing was intensified, and public health facilities were equipped with more infrastructure and supplies. Districts with more than 10% TPR were monitored very closely. Strict restrictions and containment measures were imposed while ensuring availability of essential services. Positive cases and their contacts were monitored closely, and resources were diverted to health care facilities in these districts. Most importantly, for early detection of cases, testing was massively upscaled and made widely available. All districts were advised to always maintain vaccination at highest possible levels. These actions were decentralized and implemented by the states with guidance from the central government.

## Contribution of TPR Based Restrictions in Reducing Transmission

**Figure 2:**
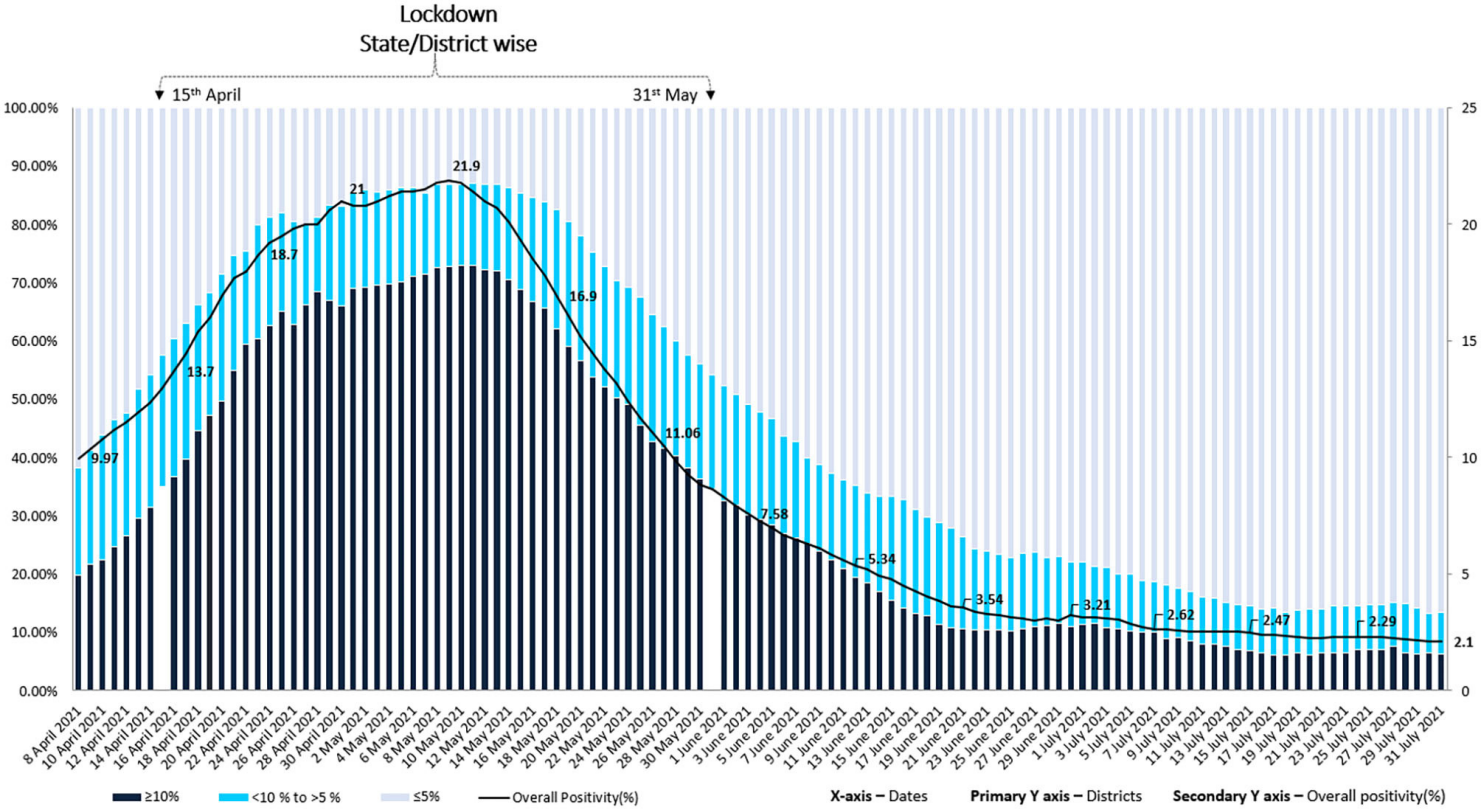
Trends of numbers of districts with <5%; ≥5 to <10% and ≥10% test positivity rates from April 1st to July 31st, 2021 depicted for all 734 districts of India. The intervention (TPR guided localized restrictions and public health action) was implemented in mid-April 2021.

TPR based restriction or relaxation strategy alongwith enforcement of other NPIs, was implemented in several parts of India from the second week of April 2021 when the disease transmission was high and national average TPR was around 10%. As described earlier, there was heterogeneity in the restrictive measures implemented across states at different time points. Most of the states relaxed restrictions by May 31st, 2021 when the TPR started declining, however some continued till middle of June. The overall national impact of TPR based restrictions was measured from April 15th to July 30th, 2021. At the outset, out of a total of 734 districts, the numbers reporting more than 10% TPR was 257 which peaked to an all-time high of 535 districts in second week of May. Thereafter, the number of districts with more than 10% TPR declined to 254,114, 50, and 46 on 31st May, 15th June, 15th July, and 31st July, respectively. This decline was accompanied by a rise in number of districts with TPR of <5% from 95 districts in second week of May to 337, 489, 628, and 635 on 31st May, 15th June, 15th July, and 31st July, respectively. Number of districts having TPR between 5 and 10% ranged from 94 to 174 from 15th April to 26th June. Thereafter a steady decline to 56 districts was observed till 15th July. The trends again did not change much after this time point (Supplementary Material). TPR trends for India from April 1st to July 31st, 2021 are depicted in Figure 2. Within 4 weeks of implementation of the TPR guided decision matrix, the number of districts with more than 10% TPR steadily declined, with simultaneous increase in number of districts having <5% TPR. The strategy helped in effective disease control even in high transmission settings within a short time span and TPR did not increase above 5% for the next few months. Our analysis is limited by the fact that we did not have data on the extent of use of various NPIs like masking, social distancing and community sensitization practiced in different parts of India.

## Gaps in Resource Planning

Implementing restrictive measures based on TPR guided tool alongwith enforcement of other NPIs, helped in bringing down TPR and reducing disease transmission within a 6–8 weeks timeframe. However, these restrictions were imposed by most of the states at the peak of transmission when TPR in most of the large states ranged between 10 and 25%, healthcare system was already overburdened and much needed institutional care was not available for many. Earmarking dedicated resources and allocation of required number of hospital beds with provision of oxygen and invasive/non-invasive ventilation facility is a key component to avert the loss of lives and appropriately manage moderate-severe cases. The home-isolation and care guidelines for asymptomatic and mild-symptomatic cases were laid down by Ministry of Health and Family Welfare, Government of India and were publicized largely (16). However, many states faltered in adequate resource mobilization and availability of hospital beds perhaps due to lack of an appropriate decision-making matrix for the projected requirements. Based on various published requirements from India and abroad, we have proposed a decision matrix to indicate the required number of hospital beds, which can be used by countries for resource allocation. We have also analyzed the resource availability and gaps in the six different Indian states studied by us.

## Assumptions and Suggested Decision Matrix

We analyzed the following parameters for suggesting a decision matrix for useful resource planning:

i Estimated number of cases that may have existed in the six Indian states against the actual numbers reported by them during the specified time period.
ii Estimated requirement of hospital beds against the actual numbers available at that time.

India has conducted four national serosurveys in May–June 2020, August–September 2020, December 2020–January 2021, and June–July 2021 wherein the community-based IgG seroprevalence was estimated in 70 districts across 21 states (17–20). The case to infection ratio (CIR) was reported as 27 in the third (19) and fourth national serosurvey (20). A sentinel cross-sectional serosurvey across 290 healthcare facilities in Karnataka was conducted in September 2020. In addition to IgG antibody estimation, active SARS-CoV-2 infections were also assessed by using real time reverse transcription polymerase chain reaction (rRTPCR) and rapid antigen test (RAT). CIR in this study ranged from 11 to 112 with overall state CIR estimated at 40 (21). CIR is calculated by dividing the estimated number of infections, as per seropositivity by the actual number of cases reported during the same time. Data on positive cases in each of the six states was extracted from the central testing database at ICMR. Thereafter, we multiplied the number of cases reported per week in each state during the peak of second wave of COVID-19 in India, by a factor of 27, as estimated in the national serosurvey. This helped us to calculate the estimated number of cases that different states may have had at that point in time.

Various studies across the world have reported a wide range of asymptomatic infections of COVID-19, varying by age (22). Earlier estimates suggested that asymptomatic and mild infections may constitute upto 80% of the cases (23). A study from India reported 91% asymptomatic infections. (24). However, large studies, meta-analysis and systematic reviews published during the last 1 year suggest that the range of asymptomatic COVID-19 infections may vary from 13 to 40% (22, 25–30). For better planning, we opted for a relatively conservative estimate of 25% asymptomatic infections to calculate the estimated number of hospital beds required during the second wave, in each Indian state. Available international data suggests that 80% of the symptomatic infections may be mild in nature (31, 32) and can be managed at home (16). The remaining 20% may be moderate to severe in nature and may require hospitalization. If data is available, a simple index to estimate the required hospital bed capacity is the Infection to Hospitalization rate (IHR), which is calculated by dividing the number of individuals who were hospitalized due to COVID-19 by the estimated number of individuals who had SARS-CoV-2 antibodies, using the seroprevalence data (33). Few published studies from other countries have estimated an IHR of 2.1 and 2.7, respectively (34, 35). Till date, India does not have any published and reliable IHR estimates for COVID-19, therefore we could not include this indicator in our assumptions. Data of 18,961 patients, published from the Indian COVID-19 clinical registry and 19,852 from a large private hospital chain in India indicates that the median duration of hospital stay in patients infected with SARS-CoV-2 in the second wave was about 7 days (36, 37). The clinical data from India, during the same time also indicates that 50–75% of the admitted patients required supplemental oxygen and of these, non-invasive/invasive ventilation was later required by about 20% patients (37). ICU care was required by about 33% of the patients (37). We have estimated the total number of hospital beds that were required at the peak of the second wave in India for improved access and availability, using the assumptions of CIR of 27; asymptomatic infection in 25% of those infected; 80% of the symptomatic individuals presenting with mild illness and not requiring hospitalization; median duration of hospitalization of 7 days; 50% of the infected patients requiring hospitalization and 20% requiring mechanical/non-mechanical ventilation. Using all this information, we calculated the requirement of hospital beds during the peak of the second wave (Table 1). A significant shortfall of hospital beds was observed in all six states of India. We could not calculate the proportionate shortfall in oxygen and ICU beds, using the above assumptions, due to absence of reliable information in public domain on availability of such beds in various states of India.

**Table 1:** Assessment of hospital bed availability in six Indian states (April 15th, 2021 to May 31st, 2021).

**S. No**.	**State**	**Total number of available COVID-19 beds**	**Average number of reported daily positives**	**Average daily COVID-19 test positivity (%)**	**Case to infection ratio (CIR)**	**Estimated true cases (ETC) (CIR × reported daily positives)**	**Estimated symptomatic positives (ESP) (75% of ETC)**	**Estimated hospital beds needed (20% of ESP)**	**Estimated no. of oxygen beds required**	**Estimated no. of invasive/non-invasive ventilator beds required**	**Estimated number of days of hospitalization**	**Actual no. of days for which hospital beds were available**	**References**
1	Delhi	21,528	15,182	19.68	27	4,09,914	3,07,436	61,487	30,744	12297	7	2.9	(38–40)
2	Andhra Pradesh	48,423	13,189	21.96	27	3,56,103	2,67,077	53,416	26,708	10683	7	1.1	(40, 41)
3	Maharashtra	2,10,091	47,609	18.87	27	1,285,443	9,64,082	1,92,817	96,408	38563	7	0.9	(40, 42)
4	Bihar	30,093	9,087	7.56	27	2,45,349	1,84,012	36,802	18,401	7360	7	1.2	(40, 43)
5	Madhya Pradesh	32,524	11,443	19.93	27	3,08,961	2,31,721	46,344	23,172	9269	7	1.4	(40, 44)
6	Assam	6,532	3,987	9.38	27	1,07,649	80,737	16,147	8,074	3229	7	2.5	(40, 45)

*Assumptions*:

*Case to Infection ratio = 27^17^*:

*1/4th of the COVID-19 cases remain asymptomatic throughout the course of illness^20^*:

*Out of the 3/4th symptomatic infections, 80% are mild in nature and can be managed at home^21^*:

*Out of the 3/4th symptomatic infections, 20% may be moderate-severe cases and may require hospitalization^21^*:

*Mean duration of hospital stay would be 7 days^25, 26^*:

*Estimated no. of beds with supplemental oxygen = 50% of the total beds^25^*:

*Estimated no. of beds with mechanical/non-mechanical ventilation = 20% of the total beds^2^*.

## Conclusions

In future, with more and more people getting vaccinated and infected with SARS-CoV-2 globally, it is expected that COVID-19 will eventually become endemic and localized outbreaks may be seen wherever susceptible population pools develop or new variants emerge. In view of this, it is imperative to formulate simple tools to guide scientific evidence based early local interventions to interrupt disease transmission. Further, such tools will also help in judiciously prioritizing healthcare measures, especially in resource limited settings. During the second wave of COVID-19 in India, 7-day rolling average of test positivity rate was used to segregate districts into high, moderate, and low transmission zones. This information became the guiding tool to impose/ease local restrictions, enforce other NPIs and implement focused public health interventions on a real-time basis. The state of Madhya Pradesh could achieve overall reduction in TPR by imposing district specific restrictions and other NPIs, guided by TPR rates. This resulted in similar outcomes as compared to Delhi and Bihar where complete lockdowns were imposed for more than a month. The observed trends suggest that pragmatic and well calibrated localized restrictions, tailored as per specific needs may achieve a decline in disease transmission comparable to drastic steps like national lockdowns. However, such decentralized decisions can only be effective if they are driven by robust local data. The WHO interim guidance on public health surveillance for COVID-19 (46) defines the modalities of surveillance in various settings, which may be challenging to implement in developing countries due to resource limitations. As per feasibility, it is critical for countries to generate local data on actual disease positivity, magnitude of testing, hospitalization trends, proportion of mild, moderate and severe infections and uptake of various NPIs to make evidence based policies. This may be achieved by setting up passive sentinel surveillance (21) or undertaking periodic community based serosurveys (47). COVID-19 hospital registries can be created to understand the outcomes of hospitalized patients, need for mechanical and non-mechanical ventilation, duration of hospital stay and fine-tuning the clinical management protocols (36). In addition, availability of a standardized global tool, adjusted against various potential confounders, will help in assessing the adequacy of the health system in the backdrop of fluctuating disease transmission dynamics.

Overall, TPR based district segregation and adoption of other NPIs appears to be an effective and pragmatic strategy for targeted restrictions and quick disease reduction even in high transmission settings. Since this strategy was adopted in India when the pandemic was already raging in various parts, reduction in disease transmission was achieved at the expense of several lives and tremendous burden on health care settings and workers. In future, it will be valuable to club TPR based intervention with enforcement of NPIs, adequate provision of hospital beds, oxygen supplies, ventilators, ICU facility, healthcare workers and essential medicines. However, this strategy has its own limitations. The actual number of tests which should be performed to accurately determine TPR is questionable. WHO recommends a minimum of one test per thousand population per week to establish a TPR of <5% (15). However, this requirement may vary in different geographical settings and disease transmission rates. The number of tests performed in different parts of India varies due to issues related to access, availability, and health seeking behavior of the community. RATs are affordable, widely used in India and account for overall 40% of the total COVID-19 tests conducted in the country (48). They are known to have low sensitivity, thereby resulting in false negatives. Districts with heavy reliance on RAT may under-report the TPR. Factors like delayed uploading of test results by certain districts/states also affects TPR interpretation. In view this, TPR must be calculated carefully and based on reliable testing data. The IHR, CIR along with robust serosurvey and clinical data indicating mean duration of hospital stay, percent requirement of oxygenation and ventilation together have the potential to be used as guiding principles for decision making and allocation of required healthcare resources. Again, since states may implement some unique control practices, such parameters may vary as per the local settings. The decision matrix proposed by us is suggestive in nature and will require fine-tuning at local level.

Using simple parameters based on local clinical, laboratory and surveillance data, generic frameworks can be developed to guide evidence-based decision making for allocation of healthcare resources. However, regional adjustments of various potential biases, considering the disease transmission dynamics, proportion of elderly people above 60 years of age, pediatric and co-morbid populations will need to be accounted for regional decision-making. Simple web-based mathematical modeling tools to predict resource requirement and possibility of future waves of COVID-19, can be developed and used for predicting and planning for future waves of the COVID-19 pandemic, by giving key inputs of state or district specific seroprevalence %, number of symptomatic cases, effectiveness of restrictions, vaccination scenarios and emergence of variants (49).

We recommend that TPR based strategy clubbed with other clinical and laboratory parameters must be adopted as an integral part of the public health strategy to monitor localized surges of COVID-19 for targeted interventions. This will ensure early disease containment with minimum social impact and economic loss. While advocating the TPR based strategy, it is crucial to ensure sustained levels of high testing rates, affordable and widely accessible molecular and rapid antigen tests with high sensitivity and specificity, equitable access in underserved and rural area and high quality testing, clinical as well as epidemiological data, to enable appropriate decision making.

## Author Contributions

NG and SR: conceptualization, data analysis, and manuscript writing. SP and BB: conceptualization and critical review. All authors contributed to the article and approved the submitted version.

## Conflict of Interest

The authors declare that the research was conducted in the absence of any commercial or financial relationships that could be construed as a potential conflict of interest.

## Publisher's Note

All claims expressed in this article are solely those of the authors and do not necessarily represent those of their affiliated organizations, or those of the publisher, the editors and the reviewers. Any product that may be evaluated in this article, or claim that may be made by its manufacturer, is not guaranteed or endorsed by the publisher.
