# Supplementary material for: Use of COVID-19 Test Positivity Rate, Epidemiological, and Clinical Tools for Guiding Targeted Public Health Interventions
Source: Front Public Health. 2022 Mar 16;10:821611. doi: 10.3389/fpubh.2022.821611 (PMC8965807; doi:10.3389/fpubh.2022.821611)
Supplement: Supplementary file 1 [file Data_Sheet_1.PDF]

Daily TPM(Tests per Million) for the period of 15th April 2021 to 31st May, 2021							
S.No.	State	Total tests	Projected Population as on 1st March,2021 (000)	Avg total tests	Total projected Population as on 1st March,2021	Popoulation in million	TPM (Tests per million)
1	ANDAMAN AND NICOBAR ISLANDS	30957	400	658.7	400000	0.4	1646.6
2	ANDHRA PRADESH	2762666	52787	58780.1	52787000	52.787	1113.5
3	ARUNACHAL PRADESH	40861	1533	869.4	1533000	1.533	567.1
4	ASSAM	1955093	35043	41597.7	35043000	35.043	1187.0
5	BIHAR	5522496	123083	117499.9	123083000	123.083	954.6
6	CHANDIGARH	156478	1208	3329.3	1208000	1.208	2756.1
7	CHHATTISGARH	2815746	29493	59909.5	29493000	29.493	2031.3
8	DADRA AND NAGAR HAVELI	16268	608	346.1	608000	0.608	569.3
9	DAMAN AND DIU	19369	469	412.1	469000	0.469	878.7
10	DELHI	3426672	20571	72907.9	20571000	20.571	3544.2
11	GOA	233063	1559	4958.8	1559000	1.559	3180.7
12	GUJARAT	5698292	69788	121240.3	69788000	69.788	1737.3
13	HARYANA	2155323	29483	45857.9	29483000	29.483	1555.4
14	HIMACHAL PRADESH	503482	7394	10712.4	7394000	7.394	1448.8
15	JAMMU AND KASHMIR	719155	13408	15301.2	13408000	13.408	1141.2
16	JHARKHAND	1847867	38471	39316.3	38471000	38.471	1022.0
17	KARNATAKA	6690375	66845	142348.4	66845000	66.845	2129.5
18	KERALA	5964168	35489	126897.2	35489000	35.489	3575.7
19	LADAKH	51700	297	1100.0	297000	0.297	3703.7
20	LAKSHADWEEP	57373	68	1220.7	68000	0.068	17951.5
21	MADHYA PRADESH	2640167	84516	56173.8	84516000	84.516	664.7
22	MAHARASHTRA	1.2E+07	124437	252229.3	124437000	124.437	2027.0
23	MANIPUR	129829	3165	2762.3	3165000	3.165	872.8
24	MEGHALAYA	78807	3288	1676.7	3288000	3.288	510.0
25	MIZORAM	123995	1216	2638.2	1216000	1.216	2169.6
26	NAGALAND	29251	2192	622.4	2192000	2.192	283.9
27	ODISHA	2168218	45696	46132.3	45696000	45.696	1009.5
28	PUDUCHERRY	187947	1571	3998.9	1571000	1.571	2545.4

29	PUNJAB	2638359	30339	56135.3	30339000	30.339	1850.3
30	RAJASTHAN	1707478	79281	36329.3	79281000	79.281	458.2
31	SIKKIM	12519	677	266.4	677000	0.677	393.4
32	TAMIL NADU	6741625	76402	143438.8	76402000	76.402	1877.4
33	TELANGANA	1886876	37725	40146.3	37725000	37.725	1064.2
34	TRIPURA	136075	4071	2895.2	4071000	4.071	711.2
35	UTTAR PRADESH	1.3E+07	230907	278577.1	230907000	230.907	1206.4
36	UTTARAKHAND	1595231	11399	33941.1	11399000	11.399	2977.5
37	WEST BENGAL	2672909	98125	56870.4	98125000	98.125	579.6
**Total**				**1880097.7**		**1363**	**1997.2**

Categorization with ≤5%; >5 to <10% and ≥10% test positivity rates from 1st April to 31st July 2021 depicted for all 734 districts of India.							
				Positivity			
S.No.	Week (Time Period)		Date	≥10%	<10 % to >5 %	≤5%	Overall Positivity(%)
1	01/04/21	07/04/21	08/04/21	145	136	453	9.97
2	02/04/21	08/04/21	09/04/21	159	143	432	10.37
3	03/04/21	09/04/21	10/04/21	164	158	412	10.78
4	04/04/21	10/04/21	11/04/21	181	160	393	11.18
5	05/04/21	11/04/21	12/04/21	195	154	385	11.49
6	06/04/21	12/04/21	13/04/21	217	162	355	11.91
7	07/04/21	13/04/21	14/04/21	231	167	336	12.35
8	08/04/21	14/04/21	15/04/21	257	165	312	12.96
9	09/04/21	15/04/21	16/04/21	269	174	291	13.7
10	10/04/21	16/04/21	17/04/21	291	171	272	14.48
11	11/04/21	17/04/21	18/04/21	327	159	248	15.38
12	12/04/21	18/04/21	19/04/21	346	155	233	16.02
13	13/04/21	19/04/21	20/04/21	364	161	209	16.93
14	14/04/21	20/04/21	21/04/21	403	145	186	17.68
15	15/04/21	21/04/21	22/04/21	436	118	180	18
16	16/04/21	22/04/21	23/04/21	443	143	148	18.7
17	17/04/21	23/04/21	24/04/21	460	136	138	19.2
18	18/04/21	24/04/21	25/04/21	478	124	132	19.5
19	19/04/21	25/04/21	26/04/21	461	130	143	19.8
20	20/04/21	26/04/21	27/04/21	486	101	147	20
21	21/04/21	27/04/21	28/04/21	502	94	138	20
22	22/04/21	28/04/21	29/04/21	491	120	123	20.6
23	23/04/21	29/04/21	30/04/21	485	125	124	21
24	24/04/21	30/04/21	01/05/21	507	120	107	20.8
25	25/04/21	01/05/21	02/05/21	508	122	104	20.8
26	26/04/21	02/05/21	03/05/21	511	117	106	21
27	27/04/21	03/05/21	04/05/21	512	118	104	21.2
28	28/04/21	04/05/21	05/05/21	515	119	100	21.4
29	29/04/21	05/05/21	06/05/21	522	111	101	21.4
30	30/04/21	06/05/21	07/05/21	525	101	108	21.5
31	01/05/21	07/05/21	08/05/21	533	105	96	21.8
32	02/05/21	08/05/21	09/05/21	534	103	97	21.9
33	03/05/21	09/05/21	10/05/21	535	102	97	21.8
34	04/05/21	10/05/21	11/05/21	535	104	95	21.4
35	05/05/21	11/05/21	12/05/21	530	107	97	21
36	06/05/21	12/05/21	13/05/21	529	108	97	20.7
37	07/05/21	13/05/21	14/05/21	517	117	100	20.1
38	08/05/21	14/05/21	15/05/21	505	121	108	19.3
39	09/05/21	15/05/21	16/05/21	490	131	113	18.5
40	10/05/21	16/05/21	17/05/21	481	135	118	17.8
41	11/05/21	17/05/21	18/05/21	455	151	128	16.9
42	12/05/21	18/05/21	19/05/21	433	157	144	16.1
43	13/05/21	19/05/21	20/05/21	415	158	161	15.2
44	14/05/21	20/05/21	21/05/21	395	157	182	14.5
45	15/05/21	21/05/21	22/05/21	382	152	200	13.8

46	16/05/21	22/05/21	23/05/21	369	147	218	13.2
47	17/05/21	23/05/21	24/05/21	361	147	226	12.4
48	18/05/21	24/05/21	25/05/21	334	161	239	11.7
49	19/05/21	25/05/21	26/05/21	313	161	260	11.06
50	20/05/21	26/05/21	27/05/21	305	153	276	10.45
51	21/05/21	27/05/21	28/05/21	295	145	294	9.8
52	22/05/21	28/05/21	29/05/21	280	143	311	9.26
53	23/05/21	29/05/21	30/05/21	266	145	323	8.82
54	24/05/21	30/05/21	31/05/21	254	143	337	8.65
55	25/05/21	31/05/21	01/06/21	239	145	350	8.31
56	26/05/21	01/06/21	02/06/21	234	139	361	7.91
57	27/05/21	02/06/21	03/06/21	221	140	373	7.58
58	28/05/21	03/06/21	04/06/21	215	136	383	7.29
59	29/05/21	04/06/21	05/06/21	208	134	392	7
60	30/05/21	05/06/21	06/06/21	198	123	413	6.68
61	31/05/21	06/06/21	07/06/21	192	121	421	6.48
62	01/06/21	07/06/21	08/06/21	185	108	441	6.28
63	02/06/21	08/06/21	09/06/21	176	109	449	6.08
64	03/06/21	09/06/21	10/06/21	165	108	461	5.82
65	04/06/21	10/06/21	11/06/21	154	111	469	5.6
66	05/06/21	11/06/21	12/06/21	143	115	476	5.34
67	06/06/21	12/06/21	13/06/21	135	114	485	5.19
68	07/06/21	13/06/21	14/06/21	124	120	490	4.92
69	08/06/21	14/06/21	15/06/21	114	131	489	4.78
70	09/06/21	15/06/21	16/06/21	104	136	494	4.5
71	10/06/21	16/06/21	17/06/21	97	131	506	4.26
72	11/06/21	17/06/21	18/06/21	94	125	515	4.03
73	12/06/21	18/06/21	19/06/21	83	128	523	3.85
74	13/06/21	19/06/21	20/06/21	79	125	530	3.62
75	14/06/21	20/06/21	21/06/21	78	116	540	3.54
76	15/06/21	21/06/21	22/06/21	76	103	555	3.39
77	16/06/21	22/06/21	23/06/21	76	100	558	3.29
78	17/06/21	23/06/21	24/06/21	76	96	562	3.22
79	18/06/21	24/06/21	25/06/21	75	92	567	3.14
80	19/06/21	25/06/21	26/06/21	77	96	561	3.11
81	20/06/21	26/06/21	27/06/21	81	93	560	3.02
82	21/06/21	27/06/21	28/06/21	82	85	567	3.09
83	22/06/21	28/06/21	29/06/21	84	84	566	3.02
84	23/06/21	29/06/21	30/06/21	80	82	572	3.21
85	24/06/21	30/06/21	01/07/21	83	79	572	3.14
86	25/06/21	01/07/21	02/07/21	85	71	578	3.13
87	26/06/21	02/07/21	03/07/21	79	76	579	3.08
88	27/06/21	03/07/21	04/07/21	77	70	587	3.03
89	28/06/21	04/07/21	05/07/21	75	71	588	2.88
90	29/06/21	05/07/21	06/07/21	73	65	596	2.72
91	30/06/21	06/07/21	07/07/21	73	64	597	2.62
92	01/07/21	07/07/21	08/07/21	65	68	601	2.6
93	02/07/21	08/07/21	09/07/21	66	63	605	2.59
94	03/07/21	09/07/21	10/07/21	63	61	610	2.54
95	04/07/21	10/07/21	11/07/21	59	58	617	2.52
96	05/07/21	11/07/21	12/07/21	58	58	618	2.54
97	06/07/21	12/07/21	13/07/21	56	55	623	2.53

98	07/07/21	13/07/21	14/07/21	51	57	626	2.51
99	08/07/21	14/07/21	15/07/21	50	56	628	2.47
100	09/07/21	15/07/21	16/07/21	47	56	631	2.41
101	10/07/21	16/07/21	17/07/21	44	60	630	2.37
102	11/07/21	17/07/21	18/07/21	45	53	636	2.34
103	12/07/21	18/07/21	19/07/21	47	54	633	2.31
104	13/07/21	19/07/21	20/07/21	45	57	632	2.27
105	14/07/21	20/07/21	21/07/21	47	55	632	2.27
106	15/07/21	21/07/21	22/07/21	48	59	627	2.29
107	16/07/21	22/07/21	23/07/21	47	59	628	2.31
108	17/07/21	23/07/21	24/07/21	52	54	628	2.29
109	18/07/21	24/07/21	25/07/21	52	56	626	2.28
110	19/07/21	25/07/21	26/07/21	52	56	626	2.3
111	20/07/21	26/07/21	27/07/21	55	56	623	2.25
112	21/07/21	27/07/21	28/07/21	48	62	624	2.18
113	22/07/21	28/07/21	29/07/21	46	58	630	2.17
114	23/07/21	29/07/21	30/07/21	47	50	637	2.1
115	24/07/21	30/07/21	31/07/21	46	53	635	2.1

Mean Test positivity rate for the period of 8th April to 31st May, 2021 of all the districts in India			
S.No	State	District	Mean TPR
1	ANDAMAN AND NICOBAR ISLANDS	NICOBARS	12.96
2	ANDAMAN AND NICOBAR ISLANDS	NORTH AND MIDDLE ANDAMAN	7.23
3	ANDAMAN AND NICOBAR ISLANDS	SOUTH ANDAMANS	4.46
4	ANDHRA PRADESH	ANANTAPUR	24.20
5	ANDHRA PRADESH	CHITTOOR	27.81
6	ANDHRA PRADESH	EAST GODAVARI	22.51
7	ANDHRA PRADESH	GUNTUR	17.63
8	ANDHRA PRADESH	KRISHNA	14.29
9	ANDHRA PRADESH	KURNOOL	20.48
10	ANDHRA PRADESH	PRAKASAM	13.15
11	ANDHRA PRADESH	SPSR NELLORE	21.49
12	ANDHRA PRADESH	SRIKAKULAM	23.26
13	ANDHRA PRADESH	VISAKHAPATANAM	27.32
14	ANDHRA PRADESH	VIZIANAGARAM	19.99
15	ANDHRA PRADESH	WEST GODAVARI	12.02
16	ANDHRA PRADESH	Y.S.R.	14.57
17	ARUNACHAL PRADESH	ANJAW	23.91
18	ARUNACHAL PRADESH	CHANGLANG	66.32
19	ARUNACHAL PRADESH	DIBANG VALLEY	38.98
20	ARUNACHAL PRADESH	EAST KAMENG	35.07
21	ARUNACHAL PRADESH	EAST SIANG	5.06
22	ARUNACHAL PRADESH	KAMLE	14.41
23	ARUNACHAL PRADESH	KRA DAADI	0.00
24	ARUNACHAL PRADESH	KURUNG KUMEY	5.24
25	ARUNACHAL PRADESH	LEPARADA	23.26
26	ARUNACHAL PRADESH	LOHIT	11.93
27	ARUNACHAL PRADESH	LONGDING	3.29
28	ARUNACHAL PRADESH	LOWER DIBANG VALLEY	26.96
29	ARUNACHAL PRADESH	LOWER SIANG	21.78
30	ARUNACHAL PRADESH	LOWER SUBANSIRI	5.95
31	ARUNACHAL PRADESH	NAMSAI	51.61
32	ARUNACHAL PRADESH	PAKKE KESSANG	20.34
33	ARUNACHAL PRADESH	PAPUM PARE	18.78
34	ARUNACHAL PRADESH	SHI YOMI	12.53
35	ARUNACHAL PRADESH	SIANG	3.07
36	ARUNACHAL PRADESH	TAWANG	28.14
37	ARUNACHAL PRADESH	TIRAP	5.21
38	ARUNACHAL PRADESH	UPPER SIANG	28.69
39	ARUNACHAL PRADESH	UPPER SUBANSIRI	38.04
40	ARUNACHAL PRADESH	WEST KAMENG	6.50
41	ARUNACHAL PRADESH	WEST SIANG	7.94
42	ASSAM	BAKSA	4.77
43	ASSAM	BARPETA	5.35

44	ASSAM	BISWANATH	5.76
45	ASSAM	BONGAIGAON	4.51
46	ASSAM	CACHAR	7.87
47	ASSAM	CHARAIDEO	6.07
48	ASSAM	CHIRANG	4.78
49	ASSAM	DARRANG	5.63
50	ASSAM	DHEMAJI	5.73
51	ASSAM	DHUBRI	5.51
52	ASSAM	DIBRUGARH	14.94
53	ASSAM	DIMA HASAO	6.46
54	ASSAM	GOALPARA	5.11
55	ASSAM	GOLAGHAT	6.49
56	ASSAM	HAILAKANDI	6.37
57	ASSAM	HOJAI	8.69
58	ASSAM	JORHAT	6.07
59	ASSAM	KAMRUP	8.84
60	ASSAM	KAMRUP METRO	13.02
61	ASSAM	KARBI ANGLONG	3.83
62	ASSAM	KARIMGANJ	4.49
63	ASSAM	KOKRAJHAR	12.34
64	ASSAM	LAKHIMPUR	7.03
65	ASSAM	MAJULI	9.65
66	ASSAM	MARIGAON	8.68
67	ASSAM	NAGAON	7.07
68	ASSAM	NALBARI	14.29
69	ASSAM	SIVASAGAR	5.30
70	ASSAM	SONITPUR	7.05
71	ASSAM	SOUTH SALMARA MANCACHAR	5.24
72	ASSAM	TINSUKIA	12.39
73	ASSAM	UDALGURI	7.55
74	ASSAM	WEST KARBI ANGLONG	3.84
75	BIHAR	ARARIA	6.30
76	BIHAR	ARWAL	9.22
77	BIHAR	AURANGABAD	18.12
78	BIHAR	BANKA	4.08
79	BIHAR	BEGUSARAI	10.17
80	BIHAR	BHAGALPUR	9.11
81	BIHAR	BHOJPUR	2.91
82	BIHAR	BUXAR	7.78
83	BIHAR	DARBHANGA	7.87
84	BIHAR	GAYA	11.58
85	BIHAR	GOPALGANJ	7.32
86	BIHAR	JAMUI	12.01
87	BIHAR	JEHANABAD	3.86
88	BIHAR	KAIMUR (BHABUA)	2.74
89	BIHAR	KATIHAR	6.32
90	BIHAR	KHAGARIA	6.27
91	BIHAR	KISHANGANJ	4.94

92	BIHAR	LAKHISARAI	7.82
93	BIHAR	MADHEPURA	7.91
94	BIHAR	MADHUBANI	9.12
95	BIHAR	MUNGER	9.27
96	BIHAR	MUZAFFARPUR	13.11
97	BIHAR	NALANDA	9.83
98	BIHAR	NAWADA	3.58
99	BIHAR	PASHCHIM CHAMPARAN	14.45
100	BIHAR	PATNA	17.36
101	BIHAR	PURBI CHAMPARAN	6.19
102	BIHAR	PURNIA	11.62
103	BIHAR	ROHTAS	5.88
104	BIHAR	SAHARSA	12.33
105	BIHAR	SAMASTIPUR	7.92
106	BIHAR	SARAN	7.09
107	BIHAR	SHEIKHPURA	11.12
108	BIHAR	SHEOHAR	7.18
109	BIHAR	SITAMARHI	5.52
110	BIHAR	SIWAN	9.17
111	BIHAR	SUPAUL	8.26
112	BIHAR	VAISHALI	8.48
113	CHANDIGARH	CHANDIGARH	20.56
114	CHHATTISGARH	BALOD	19.10
115	CHHATTISGARH	BALODA BAZAR	30.23
116	CHHATTISGARH	BALRAMPUR	19.99
117	CHHATTISGARH	BASTAR	10.97
118	CHHATTISGARH	BEMETARA	27.48
119	CHHATTISGARH	BIJAPUR	4.40
120	CHHATTISGARH	BILASPUR	29.15
121	CHHATTISGARH	DANTEWADA	10.11
122	CHHATTISGARH	DHAMTARI	23.03
123	CHHATTISGARH	DURG	32.17
124	CHHATTISGARH	GARIYABAND	27.09
125	CHHATTISGARH	GAURELLA PENDRA MARWAHI	14.76
126	CHHATTISGARH	JANJGIR CHAMPA	29.05
127	CHHATTISGARH	JASHPUR	26.23
128	CHHATTISGARH	KABIRDHAM	33.25
129	CHHATTISGARH	KANKER	11.70
130	CHHATTISGARH	KONDAGAON	10.89
131	CHHATTISGARH	KORBA	24.00
132	CHHATTISGARH	KOREA	20.02
133	CHHATTISGARH	MAHASAMUND	27.94
134	CHHATTISGARH	MUNGELI	24.73
135	CHHATTISGARH	NARAYANPUR	6.42
136	CHHATTISGARH	RAIGARH	26.38
137	CHHATTISGARH	RAIPUR	30.97
138	CHHATTISGARH	RAJNANDGAON	20.21
139	CHHATTISGARH	SUKMA	6.39

140	CHHATTISGARH	SURAJPUR	23.57
141	CHHATTISGARH	SURGUJA	15.25
142	DADRA AND NAGAR HAVELI	DADRA AND NAGAR HAVELI	7.28
143	DAMAN AND DIU	DAMAN	3.50
144	DAMAN AND DIU	DIU	0.99
145	DELHI	CENTRAL	15.67
146	DELHI	EAST	18.21
147	DELHI	NEW DELHI	22.32
148	DELHI	NORTH	15.95
149	DELHI	NORTH EAST	12.14
150	DELHI	NORTH WEST	20.69
151	DELHI	SHAHDARA	11.09
152	DELHI	SOUTH	18.49
153	DELHI	SOUTH EAST	16.47
154	DELHI	SOUTH WEST	22.71
155	DELHI	WEST	25.58
156	GOA	NORTH GOA	37.38
157	GOA	SOUTH GOA	35.21
158	GUJARAT	AHMADABAD	8.96
159	GUJARAT	AMRELI	12.68
160	GUJARAT	ANAND	7.16
161	GUJARAT	ARVALLI	11.57
162	GUJARAT	BANAS KANTHA	6.66
163	GUJARAT	BHARUCH	13.42
164	GUJARAT	BHAVNAGAR	11.64
165	GUJARAT	BOTAD	2.82
166	GUJARAT	CHHOTAUDEPUR	5.66
167	GUJARAT	DANG	5.13
168	GUJARAT	DEVBHUMI DWARKA	6.20
169	GUJARAT	DOHAD	8.98
170	GUJARAT	GANDHINAGAR	9.29
171	GUJARAT	GIR SOMNATH	11.07
172	GUJARAT	JAMNAGAR	8.76
173	GUJARAT	JUNAGADH	8.13
174	GUJARAT	KACHCHH	10.02
175	GUJARAT	KHEDA	7.78
176	GUJARAT	MAHESANA	13.95
177	GUJARAT	MAHISAGAR	6.37
178	GUJARAT	MORBI	3.29
179	GUJARAT	NARMADA	12.60
180	GUJARAT	NAVSARI	10.10
181	GUJARAT	PANCH MAHALS	12.20
182	GUJARAT	PATAN	15.37
183	GUJARAT	PORBANDAR	8.59
184	GUJARAT	RAJKOT	13.88
185	GUJARAT	SABAR KANTHA	7.20
186	GUJARAT	SURAT	3.84
187	GUJARAT	SURENDRANAGAR	8.71

188	GUJARAT	TAPI	16.11
189	GUJARAT	VADODARA	12.16
190	GUJARAT	VALSAD	10.23
191	HARYANA	AMBALA	20.71
192	HARYANA	BHIWANI	30.27
193	HARYANA	CHARKI DADRI	17.09
194	HARYANA	FARIDABAD	30.55
195	HARYANA	FATEHABAD	22.78
196	HARYANA	GURUGRAM	24.96
197	HARYANA	HISAR	24.32
198	HARYANA	JHAJJAR	20.15
199	HARYANA	JIND	27.51
200	HARYANA	KAITHAL	11.31
201	HARYANA	KARNAL	25.59
202	HARYANA	KURUKSHETRA	17.28
203	HARYANA	MAHENDRAGARH	22.85
204	HARYANA	NUH	23.02
205	HARYANA	PALWAL	26.88
206	HARYANA	PANCHKULA	23.82
207	HARYANA	PANIPAT	38.51
208	HARYANA	REWARI	29.91
209	HARYANA	ROHTAK	29.64
210	HARYANA	SIRSA	13.19
211	HARYANA	SONIPAT	36.89
212	HARYANA	YAMUNANAGAR	30.48
213	HIMACHAL PRADESH	BILASPUR	12.91
214	HIMACHAL PRADESH	CHAMBA	10.22
215	HIMACHAL PRADESH	HAMIRPUR	13.05
216	HIMACHAL PRADESH	KANGRA	19.85
217	HIMACHAL PRADESH	KINNAUR	11.57
218	HIMACHAL PRADESH	KULLU	15.74
219	HIMACHAL PRADESH	LAHUL AND SPITI	37.54
220	HIMACHAL PRADESH	MANDI	22.24
221	HIMACHAL PRADESH	SHIMLA	13.59
222	HIMACHAL PRADESH	SIRMAUR	23.84
223	HIMACHAL PRADESH	SOLAN	22.64
224	HIMACHAL PRADESH	UNA	17.21
225	JAMMU AND KASHMIR	ANANTNAG	10.73
226	JAMMU AND KASHMIR	BANDIPORA	4.90
227	JAMMU AND KASHMIR	BARAMULLA	7.25
228	JAMMU AND KASHMIR	BUDGAM	13.09
229	JAMMU AND KASHMIR	DODA	3.29
230	JAMMU AND KASHMIR	GANDERBAL	7.41
231	JAMMU AND KASHMIR	JAMMU	23.34
232	JAMMU AND KASHMIR	KATHUA	6.45
233	JAMMU AND KASHMIR	KISHTWAR	3.34
234	JAMMU AND KASHMIR	KULGAM	6.91
235	JAMMU AND KASHMIR	KUPWARA	8.81

236	JAMMU AND KASHMIR	POONCH	2.21
237	JAMMU AND KASHMIR	PULWAMA	12.46
238	JAMMU AND KASHMIR	RAJOURI	3.73
239	JAMMU AND KASHMIR	RAMBAN	2.93
240	JAMMU AND KASHMIR	REASI	11.22
241	JAMMU AND KASHMIR	SAMBA	8.16
242	JAMMU AND KASHMIR	SHOPIAN	3.60
243	JAMMU AND KASHMIR	SRINAGAR	14.55
244	JAMMU AND KASHMIR	UDHAMPUR	10.53
245	JHARKHAND	BOKARO	12.35
246	JHARKHAND	CHATRA	9.78
247	JHARKHAND	DEOGHAR	11.42
248	JHARKHAND	DHANBAD	5.17
249	JHARKHAND	DUMKA	6.36
250	JHARKHAND	EAST SINGHBUM	13.59
251	JHARKHAND	GARHWA	8.68
252	JHARKHAND	GIRIDIH	6.04
253	JHARKHAND	GODDA	10.13
254	JHARKHAND	GUMLA	16.01
255	JHARKHAND	HAZARIBAGH	13.77
256	JHARKHAND	JAMTARA	12.45
257	JHARKHAND	KHUNTI	8.16
258	JHARKHAND	KODERMA	14.35
259	JHARKHAND	LATEHAR	11.92
260	JHARKHAND	LOHARDAGA	15.14
261	JHARKHAND	PAKUR	2.66
262	JHARKHAND	PALAMU	9.64
263	JHARKHAND	RAMGARH	14.38
264	JHARKHAND	RANCHI	23.21
265	JHARKHAND	SAHEBGANJ	5.62
266	JHARKHAND	SARAIKELA KHARSAWAN	6.20
267	JHARKHAND	SIMDEGA	9.34
268	JHARKHAND	WEST SINGHBHUM	10.42
269	KARNATAKA	BAGALKOTE	13.48
270	KARNATAKA	BALLARI	25.65
271	KARNATAKA	BELAGAVI	18.19
272	KARNATAKA	BENGALURU RURAL	21.35
273	KARNATAKA	BENGALURU URBAN	21.98
274	KARNATAKA	BIDAR	9.73
275	KARNATAKA	CHAMARAJANAGARA	23.13
276	KARNATAKA	CHIKKABALLAPURA	17.14
277	KARNATAKA	CHIKKAMAGALURU	21.71
278	KARNATAKA	CHITRADURGA	9.72
279	KARNATAKA	DAKSHINA KANNADA	19.35
280	KARNATAKA	DAVANGERE	14.25
281	KARNATAKA	DHARWAD	20.61
282	KARNATAKA	GADAG	14.75
283	KARNATAKA	HASSAN	27.14

284	KARNATAKA	HAVERI	8.92
285	KARNATAKA	KALABURAGI	19.50
286	KARNATAKA	KODAGU	20.28
287	KARNATAKA	KOLAR	23.44
288	KARNATAKA	KOPPAL	21.76
289	KARNATAKA	MANDYA	18.17
290	KARNATAKA	MYSURU	27.71
291	KARNATAKA	RAICHUR	20.21
292	KARNATAKA	RAMANAGARA	18.41
293	KARNATAKA	SHIVAMOGGA	22.83
294	KARNATAKA	TUMAKURU	26.44
295	KARNATAKA	UDUPI	20.78
296	KARNATAKA	UTTARA KANNADA	24.84
297	KARNATAKA	VIJAYAPURA	14.39
298	KARNATAKA	YADGIR	16.24
299	KERALA	ALAPPUZHA	20.23
300	KERALA	ERNAKULAM	21.48
301	KERALA	IDUKKI	18.74
302	KERALA	KANNUR	21.05
303	KERALA	KASARAGOD	20.48
304	KERALA	KOLLAM	16.70
305	KERALA	KOTTAYAM	22.25
306	KERALA	KOZHIKODE	20.74
307	KERALA	MALAPPURAM	25.27
308	KERALA	PALAKKAD	21.65
309	KERALA	PATHANAMTHITTA	18.02
310	KERALA	THIRUVANANTHAPURAM	20.17
311	KERALA	THRISSUR	21.33
312	KERALA	WAYANAD	17.86
313	LADAKH	KARGIL	4.51
314	LADAKH	LEH LADAKH	19.39
315	LAKSHADWEEP	LAKSHADWEEP DISTRICT	10.75
316	MADHYA PRADESH	AGAR MALWA	14.88
317	MADHYA PRADESH	ALIRAJPUR	11.09
318	MADHYA PRADESH	ANUPPUR	30.24
319	MADHYA PRADESH	ASHOKNAGAR	17.10
320	MADHYA PRADESH	BALAGHAT	24.05
321	MADHYA PRADESH	BARWANI	17.12
322	MADHYA PRADESH	BETUL	25.19
323	MADHYA PRADESH	BHIND	9.20
324	MADHYA PRADESH	BHOPAL	24.48
325	MADHYA PRADESH	BURHANPUR	3.27
326	MADHYA PRADESH	CHHATARPUR	27.55
327	MADHYA PRADESH	CHHINDWARA	10.31
328	MADHYA PRADESH	DAMOH	20.58
329	MADHYA PRADESH	DATIA	22.05
330	MADHYA PRADESH	DEWAS	22.35
331	MADHYA PRADESH	DHAR	25.01

332	MADHYA PRADESH	DINDORI	16.66
333	MADHYA PRADESH	EAST NIMAR	4.67
334	MADHYA PRADESH	GUNA	9.74
335	MADHYA PRADESH	GWALIOR	23.30
336	MADHYA PRADESH	HARDA	15.55
337	MADHYA PRADESH	HOSHANGABAD	24.93
338	MADHYA PRADESH	INDORE	30.37
339	MADHYA PRADESH	JABALPUR	31.82
340	MADHYA PRADESH	JHABUA	15.88
341	MADHYA PRADESH	KATNI	29.26
342	MADHYA PRADESH	KHARGONE	18.19
343	MADHYA PRADESH	MANDLA	18.18
344	MADHYA PRADESH	MANDSAUR	18.98
345	MADHYA PRADESH	MORENA	15.22
346	MADHYA PRADESH	NARSINGHPUR	21.52
347	MADHYA PRADESH	NEEMUCH	17.05
348	MADHYA PRADESH	NIWARI	15.62
349	MADHYA PRADESH	PANNA	24.77
350	MADHYA PRADESH	RAISEN	25.06
351	MADHYA PRADESH	RAJGARH	20.34
352	MADHYA PRADESH	RATLAM	22.65
353	MADHYA PRADESH	REWA	22.71
354	MADHYA PRADESH	SAGAR	26.14
355	MADHYA PRADESH	SATNA	21.57
356	MADHYA PRADESH	SEHORE	16.64
357	MADHYA PRADESH	SEONI	24.37
358	MADHYA PRADESH	SHAHDOL	33.79
359	MADHYA PRADESH	SHAJAPUR	18.67
360	MADHYA PRADESH	SHEOPUR	11.69
361	MADHYA PRADESH	SHIVPURI	23.89
362	MADHYA PRADESH	SIDHI	26.34
363	MADHYA PRADESH	SINGRAULI	27.58
364	MADHYA PRADESH	TIKAMGARH	18.29
365	MADHYA PRADESH	UJJAIN	21.70
366	MADHYA PRADESH	UMARIA	26.93
367	MADHYA PRADESH	VIDISHA	34.32
368	MAHARASHTRA	AHMEDNAGAR	30.32
369	MAHARASHTRA	AKOLA	15.81
370	MAHARASHTRA	AMRAVATI	18.53
371	MAHARASHTRA	AURANGABAD	9.33
372	MAHARASHTRA	BEED	24.61
373	MAHARASHTRA	BHANDARA	17.24
374	MAHARASHTRA	BULDHANA	27.99
375	MAHARASHTRA	CHANDRAPUR	28.24
376	MAHARASHTRA	DHULE	12.56
377	MAHARASHTRA	GADCHIROLI	20.38
378	MAHARASHTRA	GONDIA	20.08
379	MAHARASHTRA	HINGOLI	31.68

380	MAHARASHTRA	JALGAON	9.42
381	MAHARASHTRA	JALNA	21.98
382	MAHARASHTRA	KOLHAPUR	18.30
383	MAHARASHTRA	LATUR	22.68
384	MAHARASHTRA	MUMBAI	12.86
385	MAHARASHTRA	MUMBAI SUBURBAN	17.46
386	MAHARASHTRA	NAGPUR	25.27
387	MAHARASHTRA	NANDED	19.35
388	MAHARASHTRA	NANDURBAR	17.92
389	MAHARASHTRA	NASHIK	29.79
390	MAHARASHTRA	OSMANABAD	27.60
391	MAHARASHTRA	PALGHAR	28.54
392	MAHARASHTRA	PARBHANI	24.05
393	MAHARASHTRA	PUNE	26.05
394	MAHARASHTRA	RAIGAD	22.82
395	MAHARASHTRA	RATNAGIRI	19.77
396	MAHARASHTRA	SANGLI	21.34
397	MAHARASHTRA	SATARA	28.77
398	MAHARASHTRA	SINDHUDURG	23.76
399	MAHARASHTRA	SOLAPUR	16.26
400	MAHARASHTRA	THANE	18.34
401	MAHARASHTRA	WARDHA	23.61
402	MAHARASHTRA	WASHIM	14.30
403	MAHARASHTRA	YAVATMAL	14.07
404	MANIPUR	BISHNUPUR	13.46
405	MANIPUR	CHANDEL	7.10
406	MANIPUR	CHURACHANDPUR	11.50
407	MANIPUR	IMPHAL EAST	19.03
408	MANIPUR	IMPHAL WEST	14.46
409	MANIPUR	JIRIBAM	6.84
410	MANIPUR	KAKCHING	17.41
411	MANIPUR	KAMJONG	3.86
412	MANIPUR	KANGPOKPI	7.83
413	MANIPUR	NONEY	17.65
414	MANIPUR	PHERZAWL	16.00
415	MANIPUR	SENAPATI	4.99
416	MANIPUR	TAMENGLONG	2.60
417	MANIPUR	TENGNOUPAL	3.80
418	MANIPUR	THOUBAL	9.91
419	MANIPUR	UKHRUL	5.41
420	MEGHALAYA	EAST GARO HILLS	5.19
421	MEGHALAYA	EAST JAINTIA HILLS	11.08
422	MEGHALAYA	EAST KHASI HILLS	19.68
423	MEGHALAYA	NORTH GARO HILLS	10.16
424	MEGHALAYA	RI BHOI	12.10
425	MEGHALAYA	SOUTH GARO HILLS	10.28
426	MEGHALAYA	SOUTH WEST GARO HILLS	6.66
427	MEGHALAYA	SOUTH WEST KHASI HILLS	5.17

428	MEGHALAYA	WEST GARO HILLS	8.55
429	MEGHALAYA	WEST JAINTIA HILLS	16.46
430	MEGHALAYA	WEST KHASI HILLS	4.12
431	MIZORAM	AIZAWL	5.96
432	MIZORAM	CHAMPHAI	1.83
433	MIZORAM	HNAHTHIAL	3.28
434	MIZORAM	KHAWZAWL	3.33
435	MIZORAM	KOLASIB	4.56
436	MIZORAM	LAWNGTLAI	10.33
437	MIZORAM	LUNGLEI	7.78
438	MIZORAM	MAMIT	2.93
439	MIZORAM	SAIHA	2.98
440	MIZORAM	SAITUAL	2.40
441	MIZORAM	SERCHHIP	6.71
442	NAGALAND	DIMAPUR	21.27
443	NAGALAND	KIPHIRE	4.74
444	NAGALAND	KOHIMA	16.32
445	NAGALAND	LONGLENG	8.01
446	NAGALAND	MOKOKCHUNG	7.73
447	NAGALAND	MON	20.09
448	NAGALAND	PEREN	11.96
449	NAGALAND	PHEK	8.15
450	NAGALAND	TUENSANG	5.75
451	NAGALAND	WOKHA	14.55
452	NAGALAND	ZUNHEBOTO	12.20
453	ODISHA	ANUGUL	21.72
454	ODISHA	BALANGIR	18.21
455	ODISHA	BALESHWAR	18.95
456	ODISHA	BARGARH	22.91
457	ODISHA	BHADRAK	21.06
458	ODISHA	BOUDH	20.10
459	ODISHA	CUTTACK	19.72
460	ODISHA	DEOGARH	12.78
461	ODISHA	DHENKANAL	13.75
462	ODISHA	GAJAPATI	11.59
463	ODISHA	GANJAM	7.24
464	ODISHA	JAGATSINGHAPUR	12.42
465	ODISHA	JAJAPUR	17.20
466	ODISHA	JHARSUGUDA	16.24
467	ODISHA	KALAHANDI	25.52
468	ODISHA	KANDHAMAL	6.27
469	ODISHA	KENDRAPARA	7.27
470	ODISHA	KENDUJHAR	15.57
471	ODISHA	KHORDHA	26.42
472	ODISHA	KORAPUT	7.31
473	ODISHA	MALKANGIRI	5.26
474	ODISHA	MAYURBHANJ	12.14
475	ODISHA	NABARANGPUR	19.27

476	ODISHA	NAYAGARH	10.91
477	ODISHA	NUAPADA	25.32
478	ODISHA	PURI	18.88
479	ODISHA	RAYAGADA	12.56
480	ODISHA	SAMBALPUR	30.41
481	ODISHA	SONEPUR	13.69
482	ODISHA	SUNDARGARH	27.61
483	PUDUCHERRY	KARAIKAL	22.75
484	PUDUCHERRY	MAHE	38.73
485	PUDUCHERRY	PONDICHERRY	30.29
486	PUDUCHERRY	YANAM	30.64
487	PUNJAB	AMRITSAR	16.21
488	PUNJAB	BARNALA	9.50
489	PUNJAB	BATHINDA	17.72
490	PUNJAB	FARIDKOT	13.36
491	PUNJAB	FATEHGARH SAHIB	11.31
492	PUNJAB	FAZILKA	15.69
493	PUNJAB	FIROZEPUR	12.74
494	PUNJAB	GURDASPUR	9.12
495	PUNJAB	HOSHIARPUR	9.03
496	PUNJAB	JALANDHAR	11.22
497	PUNJAB	KAPURTHALA	7.45
498	PUNJAB	LUDHIANA	12.26
499	PUNJAB	MANSA	15.65
500	PUNJAB	MOGA	11.48
501	PUNJAB	PATHANKOT	13.50
502	PUNJAB	PATIALA	12.06
503	PUNJAB	RUPNAGAR	13.18
504	PUNJAB	S.A.S NAGAR	20.34
505	PUNJAB	SANGRUR	8.14
506	PUNJAB	SHAHID BHAGAT SINGH NAGAR	5.49
507	PUNJAB	SRI MUKTSAR SAHIB	15.05
508	PUNJAB	TARN TARAN	7.38
509	RAJASTHAN	AJMER	2.21
510	RAJASTHAN	ALWAR	20.86
511	RAJASTHAN	BANSWARA	13.16
512	RAJASTHAN	BARAN	16.96
513	RAJASTHAN	BARMER	11.91
514	RAJASTHAN	BHARATPUR	25.20
515	RAJASTHAN	BHILWARA	20.20
516	RAJASTHAN	BIKANER	34.93
517	RAJASTHAN	BUNDI	11.73
518	RAJASTHAN	CHITTORGARH	21.77
519	RAJASTHAN	CHURU	14.45
520	RAJASTHAN	DAUSA	22.48
521	RAJASTHAN	DHOLPUR	25.65
522	RAJASTHAN	DUNGARPUR	2.64
523	RAJASTHAN	GANGANAGAR	21.28

524	RAJASTHAN	HANUMANGARH	22.54
525	RAJASTHAN	JAIPUR	19.42
526	RAJASTHAN	JAISALMER	15.98
527	RAJASTHAN	JALORE	2.05
528	RAJASTHAN	JHALAWAR	18.60
529	RAJASTHAN	JHUNJHUNU	18.17
530	RAJASTHAN	JODHPUR	19.86
531	RAJASTHAN	KARAULI	10.26
532	RAJASTHAN	KOTA	27.42
533	RAJASTHAN	NAGAUR	12.80
534	RAJASTHAN	PALI	14.90
535	RAJASTHAN	PRATAPGARH	27.47
536	RAJASTHAN	RAJSAMAND	30.62
537	RAJASTHAN	SAWAI MADHOPUR	16.07
538	RAJASTHAN	SIKAR	12.01
539	RAJASTHAN	SIROHI	16.50
540	RAJASTHAN	TONK	12.62
541	RAJASTHAN	UDAIPUR	34.12
542	SIKKIM	EAST DISTRICT	24.02
543	SIKKIM	NORTH DISTRICT	24.11
544	SIKKIM	SOUTH DISTRICT	17.61
545	SIKKIM	WEST DISTRICT	14.01
546	TAMIL NADU	ARIYALUR	9.79
547	TAMIL NADU	CHENGALPATTU	15.99
548	TAMIL NADU	CHENNAI	24.08
549	TAMIL NADU	COIMBATORE	23.03
550	TAMIL NADU	CUDDALORE	15.37
551	TAMIL NADU	DHARMAPURI	13.60
552	TAMIL NADU	DINDIGUL	18.47
553	TAMIL NADU	ERODE	18.31
554	TAMIL NADU	KALLAKURICHI	12.95
555	TAMIL NADU	KANCHIPURAM	13.12
556	TAMIL NADU	KANNIYAKUMARI	14.33
557	TAMIL NADU	KARUR	13.50
558	TAMIL NADU	KRISHNAGIRI	24.83
559	TAMIL NADU	MADURAI	11.32
560	TAMIL NADU	NAGAPATTINAM	24.31
561	TAMIL NADU	NAMAKKAL	14.94
562	TAMIL NADU	PERAMBALUR	7.66
563	TAMIL NADU	PUDUKKOTTAI	7.57
564	TAMIL NADU	RAMANATHAPURAM	13.32
565	TAMIL NADU	RANIPET	33.33
566	TAMIL NADU	SALEM	14.19
567	TAMIL NADU	SIVAGANGA	8.35
568	TAMIL NADU	TENKASI	14.31
569	TAMIL NADU	THANJAVUR	13.38
570	TAMIL NADU	THE NILGIRIS	10.65
571	TAMIL NADU	THENI	19.23

572	TAMIL NADU	THIRUVALLUR	14.51
573	TAMIL NADU	THIRUVARUR	12.04
574	TAMIL NADU	TIRUCHIRAPPALLI	14.61
575	TAMIL NADU	TIRUNELVELI	15.93
576	TAMIL NADU	TIRUPATHUR	13.04
577	TAMIL NADU	TIRUPPUR	20.35
578	TAMIL NADU	TIRUVANNAMALAI	21.17
579	TAMIL NADU	TUTICORIN	19.62
580	TAMIL NADU	VELLORE	13.72
581	TAMIL NADU	VILLUPURAM	15.42
582	TAMIL NADU	VIRUDHUNAGAR	17.09
583	TELANGANA	ADILABAD	5.72
584	TELANGANA	BHADRADRI KOTHAGUDEM	0.89
585	TELANGANA	HYDERABAD	5.19
586	TELANGANA	JAGITIAL	0.84
587	TELANGANA	JANGOAN	2.10
588	TELANGANA	JAYASHANKAR BHUPALAPALLY	0.03
589	TELANGANA	JOGULAMBA GADWAL	0.00
590	TELANGANA	KAMAREDDY	1.71
591	TELANGANA	KARIMNAGAR	3.03
592	TELANGANA	KHAMMAM	5.74
593	TELANGANA	KUMURAM BHEEM ASIFABAD	0.00
594	TELANGANA	MAHABUBABAD	1.71
595	TELANGANA	MAHABUBNAGAR	3.27
596	TELANGANA	MANCHERIAL	1.03
597	TELANGANA	MEDAK	4.61
598	TELANGANA	MEDCHAL MALKAJGIRI	2.31
599	TELANGANA	MULUGU	0.01
600	TELANGANA	NAGARKURNOOL	1.48
601	TELANGANA	NALGONDA	4.18
602	TELANGANA	NARAYANPET	0.02
603	TELANGANA	NIRMAL	3.92
604	TELANGANA	NIZAMABAD	6.10
605	TELANGANA	PEDDAPALLI	1.41
606	TELANGANA	RAJANNA SIRCILLA	4.98
607	TELANGANA	RANGA REDDY	5.19
608	TELANGANA	SANGAREDDY	1.15
609	TELANGANA	SIDDIPET	2.24
610	TELANGANA	SURYAPET	0.43
611	TELANGANA	VIKARABAD	5.51
612	TELANGANA	WANAPARTHY	0.02
613	TELANGANA	WARANGAL RURAL	3.43
614	TELANGANA	WARANGAL URBAN	1.82
615	TELANGANA	YADADRI BHUVANAGIRI	2.30
616	TRIPURA	DHALAI	10.25
617	TRIPURA	GOMATI	4.43

618	TRIPURA	KHOWAI	5.37
619	TRIPURA	NORTH TRIPURA	2.26
620	TRIPURA	SEPAHIJALA	3.86
621	TRIPURA	SOUTH TRIPURA	4.36
622	TRIPURA	UNAKOTI	7.57
623	TRIPURA	WEST TRIPURA	14.74
624	UTTAR PRADESH	AGRA	6.94
625	UTTAR PRADESH	ALIGARH	5.18
626	UTTAR PRADESH	AMBEDKAR NAGAR	4.70
627	UTTAR PRADESH	AMETHI	6.62
628	UTTAR PRADESH	AMROHA	13.76
629	UTTAR PRADESH	AURAIYA	7.91
630	UTTAR PRADESH	AYODHYA	6.55
631	UTTAR PRADESH	AZAMGARH	10.20
632	UTTAR PRADESH	BAGHPAT	7.06
633	UTTAR PRADESH	BAHRAICH	7.26
634	UTTAR PRADESH	BALLIA	10.19
635	UTTAR PRADESH	BALRAMPUR	6.63
636	UTTAR PRADESH	BANDA	7.01
637	UTTAR PRADESH	BARABANKI	8.97
638	UTTAR PRADESH	BAREILLY	15.47
639	UTTAR PRADESH	BASTI	4.79
640	UTTAR PRADESH	BHADOHI	6.20
641	UTTAR PRADESH	BIJNOR	8.61
642	UTTAR PRADESH	BUDAUN	8.91
643	UTTAR PRADESH	BULANDSHAHR	4.53
644	UTTAR PRADESH	CHANDAULI	15.22
645	UTTAR PRADESH	CHITRAKOOT	9.13
646	UTTAR PRADESH	DEORIA	6.19
647	UTTAR PRADESH	ETAH	9.27
648	UTTAR PRADESH	ETAWAH	8.70
649	UTTAR PRADESH	FARRUKHABAD	7.05
650	UTTAR PRADESH	FATEHPUR	2.33
651	UTTAR PRADESH	FIROZABAD	3.15
652	UTTAR PRADESH	GAUTAM BUDDHA NAGAR	18.12
653	UTTAR PRADESH	GHAZIABAD	19.84
654	UTTAR PRADESH	GHAZIPUR	15.56
655	UTTAR PRADESH	GONDA	4.26
656	UTTAR PRADESH	GORAKHPUR	8.45
657	UTTAR PRADESH	HAMIRPUR	5.12
658	UTTAR PRADESH	HAPUR	10.39
659	UTTAR PRADESH	HARDOI	6.00
660	UTTAR PRADESH	HATHRAS	1.47
661	UTTAR PRADESH	JALAUN	7.02
662	UTTAR PRADESH	JAUNPUR	10.67
663	UTTAR PRADESH	JHANSI	11.47
664	UTTAR PRADESH	KANNAUJ	6.60
665	UTTAR PRADESH	KANPUR DEHAT	5.13

666	UTTAR PRADESH	KANPUR NAGAR	10.95
667	UTTAR PRADESH	KASGANJ	3.19
668	UTTAR PRADESH	KAUSHAMBI	3.02
669	UTTAR PRADESH	KHERI	12.05
670	UTTAR PRADESH	KUSHI NAGAR	5.74
671	UTTAR PRADESH	LALITPUR	8.66
672	UTTAR PRADESH	LUCKNOW	10.94
673	UTTAR PRADESH	MAHARAJGANJ	4.90
674	UTTAR PRADESH	MAHOBA	3.98
675	UTTAR PRADESH	MAINPURI	6.26
676	UTTAR PRADESH	MATHURA	11.30
677	UTTAR PRADESH	MAU	6.07
678	UTTAR PRADESH	MEERUT	12.08
679	UTTAR PRADESH	MIRZAPUR	9.11
680	UTTAR PRADESH	MORADABAD	13.59
681	UTTAR PRADESH	MUZAFFARNAGAR	12.97
682	UTTAR PRADESH	PILIBHIT	6.93
683	UTTAR PRADESH	PRATAPGARH	8.22
684	UTTAR PRADESH	PRAYAGRAJ	10.24
685	UTTAR PRADESH	RAE BARELI	8.81
686	UTTAR PRADESH	RAMPUR	7.73
687	UTTAR PRADESH	SAHARANPUR	10.30
688	UTTAR PRADESH	SAMBHAL	6.11
689	UTTAR PRADESH	SANT KABEER NAGAR	5.47
690	UTTAR PRADESH	SHAHJAHANPUR	7.31
691	UTTAR PRADESH	SHAMLI	7.51
692	UTTAR PRADESH	SHRAVASTI	3.70
693	UTTAR PRADESH	SIDDHARTH NAGAR	4.85
694	UTTAR PRADESH	SITAPUR	5.70
695	UTTAR PRADESH	SONBHADRA	8.76
696	UTTAR PRADESH	SULTANPUR	8.39
697	UTTAR PRADESH	UNNAO	6.28
698	UTTAR PRADESH	VARANASI	17.28
699	UTTARAKHAND	ALMORA	13.05
700	UTTARAKHAND	BAGESHWAR	8.20
701	UTTARAKHAND	CHAMOLI	13.41
702	UTTARAKHAND	CHAMPAWAT	12.10
703	UTTARAKHAND	DEHRADUN	17.24
704	UTTARAKHAND	HARIDWAR	6.19
705	UTTARAKHAND	NAINITAL	21.99
706	UTTARAKHAND	PAURI GARHWAL	17.63
707	UTTARAKHAND	PITHORAGARH	13.69
708	UTTARAKHAND	RUDRA PRAYAG	14.88
709	UTTARAKHAND	TEHRI GARHWAL	17.80
710	UTTARAKHAND	UDAM SINGH NAGAR	15.03
711	UTTARAKHAND	UTTAR KASHI	11.93
712	WEST BENGAL	24 PARAGANAS NORTH	37.11
713	WEST BENGAL	24 PARAGANAS SOUTH	22.60

714	WEST BENGAL	ALIPURDUAR	9.10
715	WEST BENGAL	BANKURA	16.50
716	WEST BENGAL	BIRBHUM	31.27
717	WEST BENGAL	COOCHBEHAR	7.12
718	WEST BENGAL	DARJEELING	23.54
719	WEST BENGAL	DINAJPUR DAKSHIN	13.75
720	WEST BENGAL	DINAJPUR UTTAR	26.93
721	WEST BENGAL	HOOGHLY	21.56
722	WEST BENGAL	HOWRAH	28.38
723	WEST BENGAL	JALPAIGURI	23.61
724	WEST BENGAL	JHARGRAM	10.67
725	WEST BENGAL	KALIMPONG	14.57
726	WEST BENGAL	KOLKATA	38.12
727	WEST BENGAL	MALDAH	24.30
728	WEST BENGAL	MEDINIPUR EAST	20.70
729	WEST BENGAL	MEDINIPUR WEST	17.86
730	WEST BENGAL	MURSHIDABAD	18.28
731	WEST BENGAL	NADIA	27.51
732	WEST BENGAL	PASCHIM BARDHAMAN	28.28
733	WEST BENGAL	PURBA BARDHAMAN	16.78
734	WEST BENGAL	PURULIA	16.64
